# Enhanced Confinement of Terahertz Surface Plasmon Polaritons in Bulk Dirac Semimetal-Insulator-Metal Waveguides

**DOI:** 10.1186/s11671-018-2686-z

**Published:** 2018-10-03

**Authors:** Yi Su, Qi Lin, Xiang Zhai, Ling-Ling Wang

**Affiliations:** Key Laboratory for Micro-Nano Optoelectronic Devices of Ministry of Education, School of Physics and Electronics, Hunan University, Changsha, 410082 China

**Keywords:** Optical materials and properties, Nanoparticles, Dirac semimetals, 73.20.Mf78.67.Bf

## Abstract

A subwavelength terahertz plasmonic waveguide based on bulk Dirac semimetal (BDS)-insulator-metal (BIM) structure is investigated, which indicates that there is an optimized frequency range with the better confinement as well as lower loss. A broadband mode confinement up to *λ*_0_/15 with a relatively low loss of 1.0 dB/*λ*_0_ can be achieved. We also show that two silicon ribbons introduced into the BIM waveguide can form a dynamically tunable filter tailoring terahertz surface plasmon polaritons in deep-subwavelength scale, which can be further exploited for the design of ultra-compact THz plasmonic devices with dynamical tunability. Our results may also provide potential applications in optical filtering.

## Background

Terahertz (THz) wave has been extremely witnessed in the past few decades for its innovative applications, such as THz imaging, bio-chemical sensing, and communications [1–3]. To improve sensing sensitivity, imaging resolution, and integration level of THz devices, confining THz wave in deep subwavelength scale is urgently desired [4–6]. Surface plasmon polaritons (SPPs), the surface electromagnetic modes stimulated by the interaction between electron in conduction band of noble metal and photons in visible wavelengths, propagate along metal-insulator interface and enable the manipulation of light beyond the classical diffraction limit [7]. Sommerfeld-Zenneck modes, analog of SPPs in visible band, can be supported by metal in THz region. Metamaterials and other artificial structures, such as periodical patches, perforated plates, and brass tubes, have been proposed to tailor this loosely bound surface waves [8–10]. Unfortunately, poor confinement, high intrinsic loss, and passive tunability of this mode have severely hindered its practical applications.

Graphene plasmons, with relatively low loss, dynamical tunability, and extreme confinement to THz waves, hold promising applications in high-resolution, ultra-compact, and dynamical tunable devices. Duan et al. propose a broadband gate-tunable graphene heterostructure to coherently generate and control terahertz plasmons with dynamical tunability and higher efficiency. A robust difference frequency signal can be generated due to the tight confinement of graphene plasmon field [11]. Duan et al. firstly investigate discrete Talbot effect in dielectric graphene plasmonic waveguide arrays at THz wavelengths, which provides a novel platform for high-resolution self-image of THz waves in nanoscale [12]. Lin et al. propose an ultra-compact plasmon-induced transparency waveguide, which promises potential applications in slow light of THz waves [13, 14]. Li et al. propose a series of functional optical filters and absorber based on 2D material plasmons, which demonstrate high-integration [15], low loss, and dynamical tunability [16–18]. From these works, we can convince that it is the extreme confinement of surface plasmons that make it possible to manipulate THz waves at deep subwavelength scale.

Recently, bulk Dirac semimetals (BDS), “3-D graphene,” is brought into focus due to its ultrahigh carrier mobility up to 9 × 10^6^ cm^2^ V^−1^ s^−1^, which is much higher than the best graphene of 2 × 10^5^ cm^2^ V^−1^ s^−1^ [19]. In general, the higher carrier mobility is, the lower intrinsic loss of plasmons would be. Furthermore, the dielectric functions of BDS can be actively tuned by changing its Fermi energy. The good news is that BDS, such as Na_3_Bi [19], Cd_3_As_2_ [20], and AlCuFe quasicrystals [21], are easier to process and more stable compared with graphene, which is expected to be a new generation of plasmonic material after graphene. However, the mode confinement of SPPs in BDS-insulator interface is not optimistic. Our recent work has investigated the manipulation of the THz SPPs in double-layer BDS sheet waveguide, which indicates that the symmetric coupling mode has better confinement than the plasmonic waveguide mode in monolayer BDS film [22]. The mode index of the symmetric mode is 1.21 at 1.0 THz with the Fermi energy of BDS *E*_F_ = 70 meV, which is still inadequate to meet the demand of manipulating THz wave in deep-subwavelength scale.

In this paper, we propose a deep-subwavelength BDS-insulator-metal (BIM) waveguide with enhanced confinement, relatively low loss, and desirable tunability. Dispersion relation, propagation loss, and filtering application of this highly confined mode are investigated. Interestingly, there is an optimized frequency range with an enhanced confinement as well as a reduced loss, which has rarely been reported in the traditional SPP mode in metal structure. A broadband mode confinement up to *λ*_0_/15 with a relatively low loss of 1.0 dB/*λ*_0_ can be achieved. Different from previously studied BDS-based structure, the mode of this BIM waveguide can be efficiently transmitted through ultra-narrow slit with width smaller than *λ*_0_/2000. By taking two silicon ribbons as reflection mirrors, a dynamically tunable optical resonator has been achieved. The resonant frequency of the resonator can be dynamically tuned by varying the Fermi energy of BDS, which may find applications in THz switching and filtering.

### Theory and Simulation

**Fig. 1:**
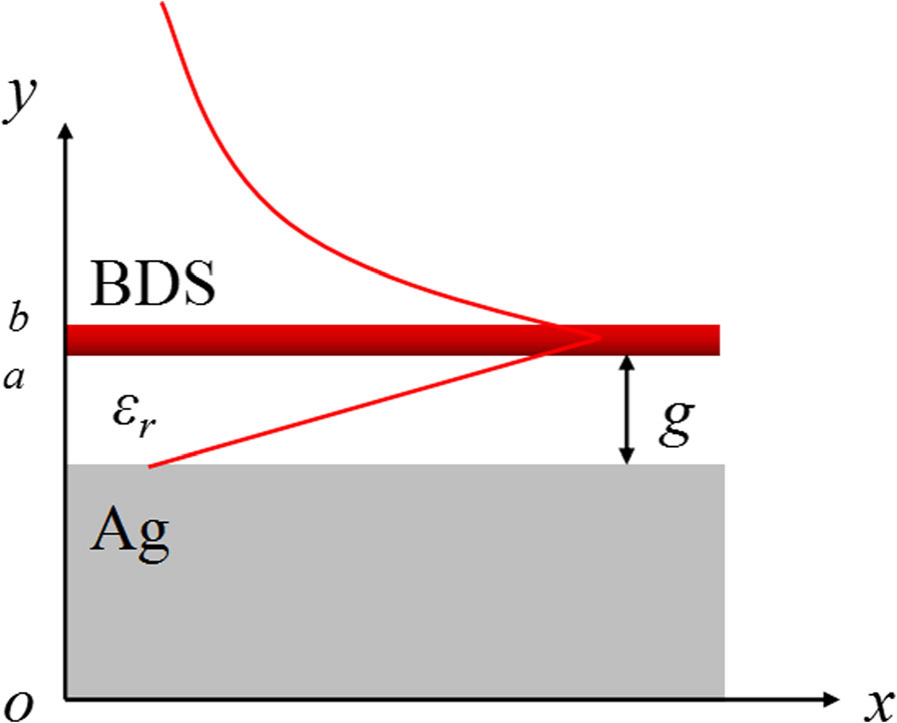
Schematic illustration of the BIM plasmonic waveguide: a monolayer BDS film is placed at a gap width *g* away from the silver substrate separated by a dielectric spacer with permittivity *ε*_*r*_. The TM-polarized plasmonic waveguide mode propagates along the *x* direction and decays along the *y* direction. Schematic depiction of the *E*_*x*_ distribution is depicted by the red line

The proposed BIM plasmonic waveguide is schematically presented in Fig. 1(a), where the monolayer BDS film with thickness of 0.2 μm is placed at a gap width *g* away from the silver substrate separated by the dielectric spacer with permittivity *ε*_*r*_. The silver substrate in THz region can be treated as a perfect electric conductor (PEC) boundary. For the TM-polarized incident light, the plasmonic waveguide mode confined in the metal-insulator interface can propagate along the *x* direction with a wavevector *k*_SPP_ and exponentially decay along the *y* direction into the free space. By combining proper boundary conditions, the wavevector *k*_SPP_ of the BIM waveguide can be obtained from the following dispersion relation: [23].

1 $$ -\frac{\varepsilon_r\sqrt{k_{\mathrm{SPP}}^2-{k}_0^2}}{\varepsilon_0\sqrt{k_{\mathrm{SPP}}^2-\frac{\varepsilon_r{k}_0^2}{\varepsilon_0}}}=\left(1+\frac{i\sigma \sqrt{k_{\mathrm{SPP}}^2-{k}_0^2}}{{\omega \varepsilon}_0}\right)\tanh \left(g\sqrt{k_{\mathrm{SPP}}^2-\frac{\varepsilon_r{k}_0^2}{\varepsilon_0}}\right), $$

where *k*_0_ is the wavevector of the incident light. By solving Eq. (1), we can obtain the effective refractive index *n*_eff_ = *k*_SPP_/*k*_0_ = Re(*n*_eff_) + *i*Im(*n*_eff_) of the proposed plasmonic waveguide. For the highly confined plasmonic waveguide modes, the real part of effective refractive index Re(*n*_eff_) roughly describe the mode confinement, while the imaginary part Im(*n*_eff_) is directly proportion to the mode propagation loss: The larger Re(*n*_eff_) is, the higher the confinement. When *g* is large enough such that tanh[*g*(*k*_SPP_^2^ − *ε*_*r*_*k*_0_^2^/*ε*_0_)] ~ 1, Eq. (1) would be reduced to the dispersion relation

2 $$ -\frac{\varepsilon_r\sqrt{k_{\mathrm{SPP}}^2-{k}_0^2}}{\varepsilon_0\sqrt{k_{\mathrm{SPP}}^2-\frac{\varepsilon_r{k}_0^2}{\varepsilon_0}}}=\left(1+\frac{i\sigma \sqrt{k_{\mathrm{SPP}}^2-{k}_0^2}}{{\omega \varepsilon}_0}\right), $$

which depicts plasmonic waveguide mode supported by a single layer of BDS alone. The complex conductivity of BDS is presented in methods Eqs (3)–(4).

## Results and Discussion

**Fig. 2:**
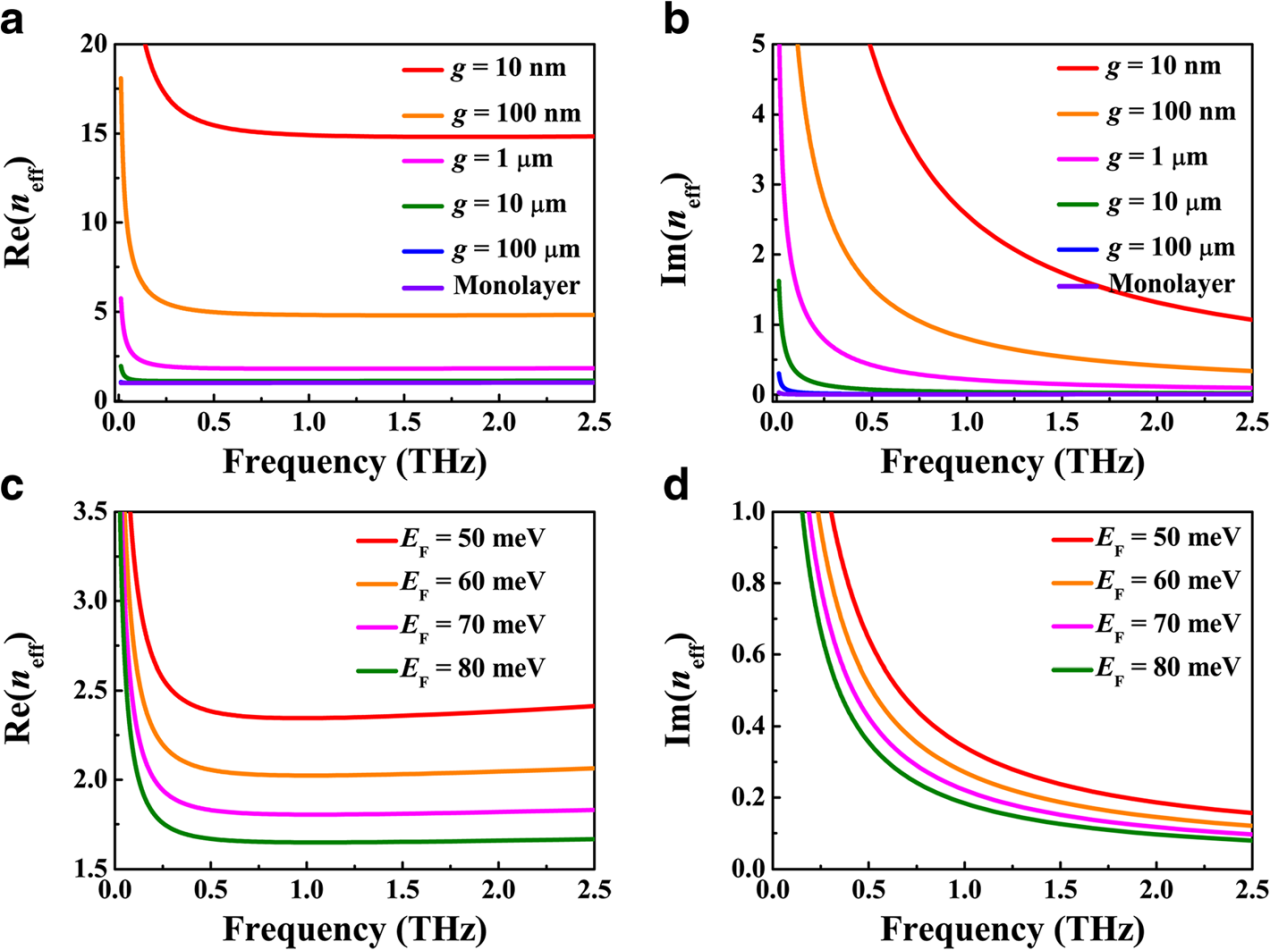
Real and imaginary parts of the effective refractive index *n*_eff_ for **a**, **b** different gap width *g*, where the Fermi energy of BDS is fixed to be *E*_F_ = 70 meV, and **c**, **d** different values of the Fermi energy *E*_F_, where the gap width is fixed at *g =* 1 μm

First, we demonstrate the dependence of the mode confinement and propagation loss of the BIM waveguide on the BDS-metal gap width *g* and Fermi energy *E*_F_. By taking *E*_F_ = 70 meV, we calculate the effective refractive indices of the SPP waveguide mode *n*_eff_ for different values of *g*, where its real and imaginary parts, Re(*n*_eff_) and Im(*n*_eff_), are plotted in Fig. 2a, b, respectively. As depicted in Fig. 2a, the curves for *g* = 10 and 100 μm merge at frequencies higher than 0.05 THz, which suggests that the plasmonic waveguide modes are so tightly confined in the BDS-insulator interface that most of the SPP fields are distributed within the scale of 10 μm and the silver would not work at such a large gap width. While the mode confinement is dramatically enhanced after the gap width *g* is gradually reduced from 1 μm, the smaller *g* researches, and the stronger confinement can be obtained. The similar tendency can be observed in the dependence of propagation loss on the gap width *g*, as depicted in Fig. 2b. On the other hand, for a fixed gap width smaller than 1 μm, Re(*n*_eff_) each initially shows a pronounced reduction to a minimum and then exhibits a gradually increasing behavior, while Im(*n*_eff_) each decreases monotonically as the frequency increases. Thus, there is an optimized frequency region where the mode confinement is strongly enhanced while the propagation loss is gradually reduced. This characteristic has rarely been observed in traditional plasmonic waveguide modes at metal-insulator interface. Figure 2c, d depicts the dependence of the mode confinement and propagation loss on the Fermi energy *E*_F_ of the BDS film, where the gap width *g* = 1 μm. Similar with the case of a monolayer and double-layer waveguide, the mode confinement and propagation loss continuously decrease with the increase of Fermi energy, which can be attributed to the enhanced metallicity and prolonged carrier relaxation time of BDS. For example, the confinement factor of plasmonic waveguide mode at 2.5 THz can be up to *λ*_0_/15, where is *λ*_0_ the incident wavelength, with a relatively low loss of 1.0 dB/*λ*_0_ when BDS-metal gap width is 10 nm and the Fermi energy is 70 meV. Therefore, relying on frameworks that have already been discussed above would increase mode confinement with a relatively low loss, which can be utilized to the design of integrated optical filters, buffers, and Mach-Zehnder interferometer.

**Fig. 3:**
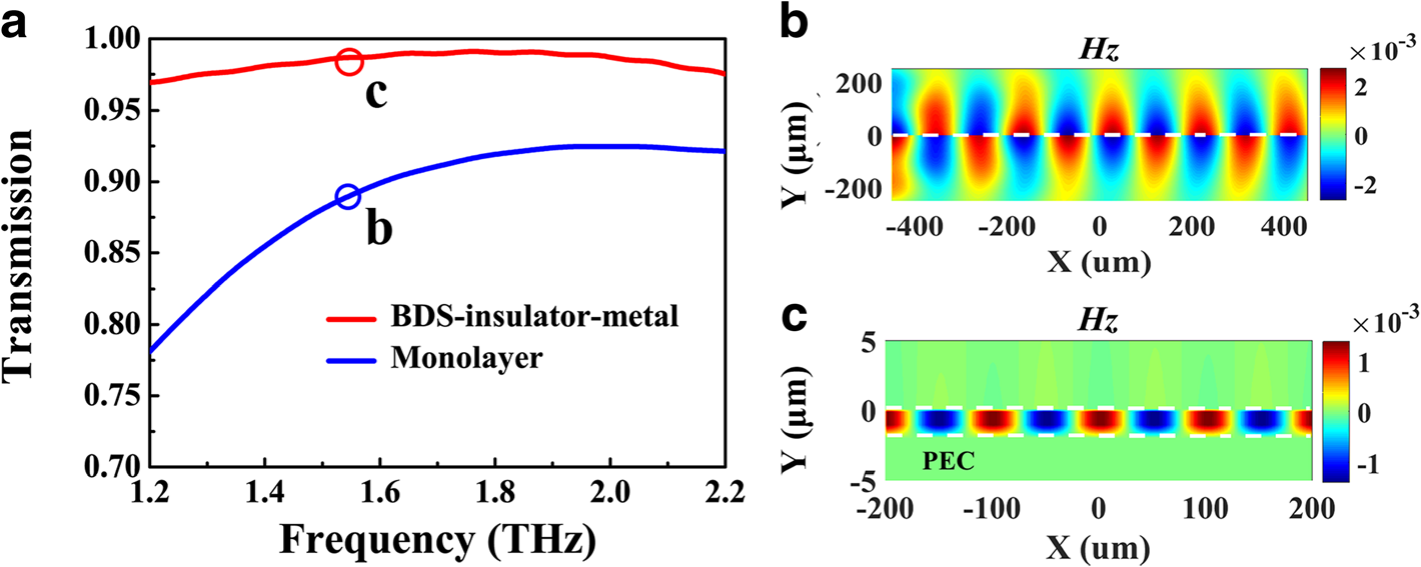
Numerical calculations of the transmission spectra (**a**) and magnetic field (*H*_z_) distributions (**b**, **c**) of BIM (red curve) and monolayer (blue curve) waveguides, where *E*_F_ = 70 meV, *g* = 50 *μ*m, and incident frequency of 1.56 THz

To examine aforementioned analysis, we perform numerical calculations about the transmission intensity and field distribution of the proposed waveguide structure. The simulation setting is described in methods. Comparing with the monolayer BDS waveguide with same Fermi energy *E*_F_ = 70 meV, the transmission intensity of the BIM waveguide at the frequency of 1.56 THz is 0.97 which is higher than that of the former, as shown in Fig. 3a, which suggests that the plasmonic waveguide mode in BIM structure suffers lower propagation loss. On the other hand, as indicated in Fig. 2a, the real part of effective refractive index of BIM at 1.56 THz Re(*n*_eff_) = 2.45, which is much higher than that of the monolayer case of 1.002. To visualize this statement, the magnetic field *Hz* distributions of these modes are presented in Fig. 3b, c. It can be clearly found that the highly confined plasmonic mode in BIM waveguide shows shorter oscillation period than that of the monolayer BDS case. In addition, most of the plasmonic field are localized in such narrow slit ~ *λ*_0_/2000, which holds promising applications in near-field enhancement for nonlinear physics.

**Fig. 4:**
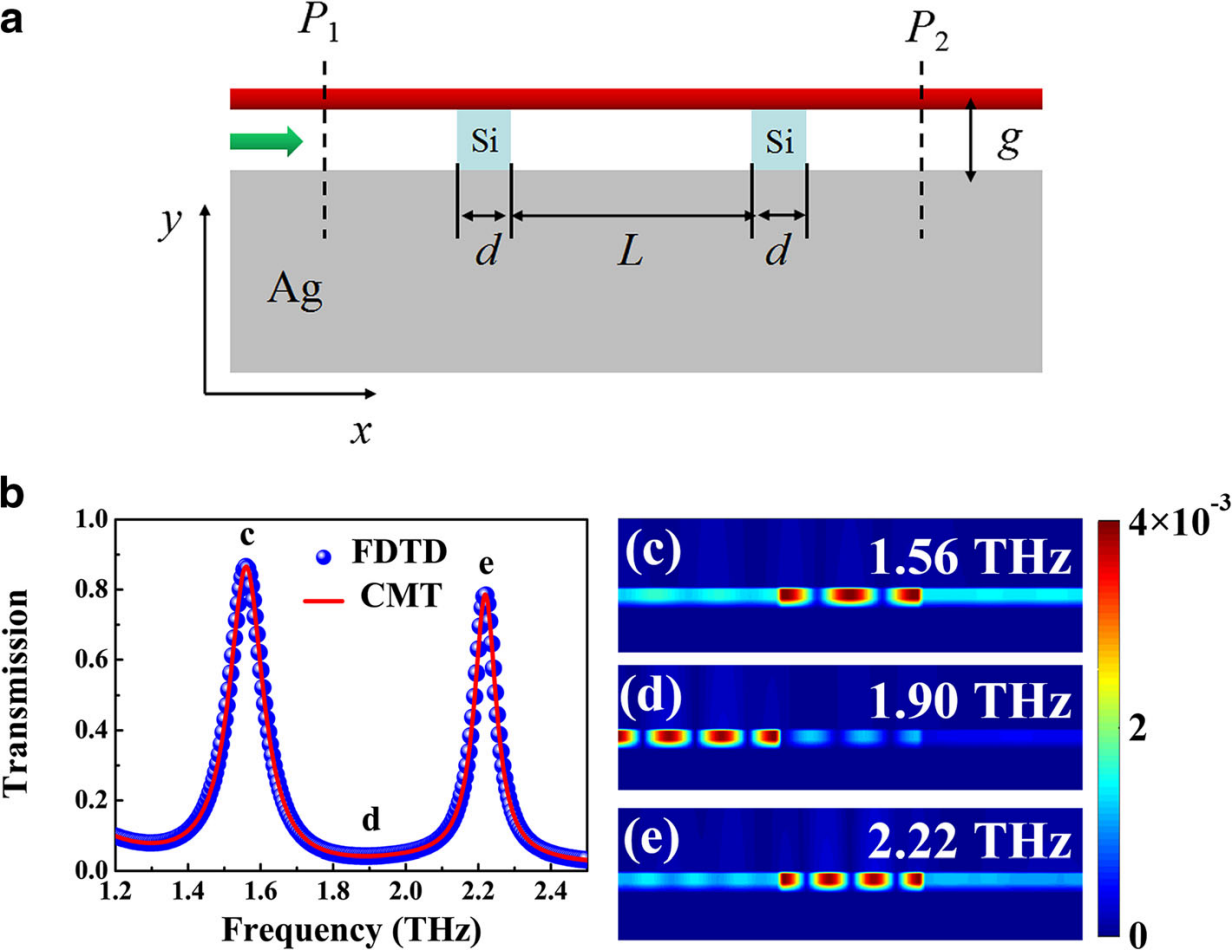
**a** Schematic of the BIM waveguide with introduced silicon ribbons. The width of each silicon ribbon is *d*, and the distance between the ribbons is *L*. **b** Numerical (blue balls) and CMT fitted (red curve) transmission spectra of the proposed structure where *g* = 1 μm, *d* = 5 μm, and *L* = 120 μm. **c**–**e** Magnetic field distributions (|*H*_*z*_|^2^) of at the incident frequencies of 1.56 (**c**), 1.90 (**d**), and 2.22 THz (**e**)

Among all the applications mentioned above, optical resonator is the essential element for tailoring THz plasmonic waveguide mode. As illustrated in Fig. 4a, two silicon (*n*_Si_ = 3.4) [24] ribbons are embedded into the dielectric spacer to form the reflective mirrors, where the propagating plasmonic wave can be reflected back and forth at the silicon-air interface forming localized standing wave resonance in the BIM region between the two silicon ribbons. Only the incident frequency satisfies the resonance condition of the standing wave, the plasmonic waves can transmit to the output of the waveguide via coupling with the designed optical resonator. Figure 4a presents the transmission spectrum of the BIM waveguide with two silicon ribbons, where two transmission peaks with FWHM (full width at half maximum) values of 0.12 and 0.09 THz can be obviously found at the frequency of 1.56 and 2.22 THz, which demonstrates novel band-pass filtering effect at terahertz region. The magnetic field distributions (|*H*_z_|^2^) of the transmission peaks are depicted in Fig. 4c, e, which implies that the BIM region sandwiched by two silicon ribbons can be regarded as a Fabry-Perot (FP) cavity. The first- and second-order resonance can be clearly found in the FP cavity. The incident plasmonic wave near the resonance frequency can be coupled into the FP cavity and then transmits through the BIM waveguide, which generates the transmission peak in the spectrum. While, for the non-resonant frequency region, the standing wave cannot be formed and thus the incident waves are prohibited in the left port of BIM waveguide, as shown in Fig. 4d. Moreover, combined with the dispersion relation of BIM waveguide, the transmission intensity can be analytically calculated by coupled mode theory (CMT) [17]:

5 $$ T\left(\omega \right)=\frac{\kappa_w^2}{{\left(\omega -{\omega}_0\right)}^2-{\left({\kappa}_w+{\kappa}_i\right)}^2}, $$

where *ω*_0_ is the resonant frequency of the FP cavity, respectively. Here, *κ*_*w*_ = *ω*_0_/(2*Q*_*w*_) and *κ*_*i*_ = *ω*_0_/(2*Q*_*i*_) are decay rates related to the waveguide coupling loss and intrinsic loss of the FP cavity, respectively. The total and intrinsic loss quality factor can be estimated by *Q*_*t*_ = *ω*_0_/FWHM, and *Q*_*oi*_ = − Re(*n*_eff_)/(2Im(*n*_eff_)), respectively. Then, the waveguide coupling loss quality factor can be obtained by subtracting the intrinsic loss from the total loss, namely, *Q*_*ei*_ *= Q*_*oi*_*Q*_*ti*_/(*Q*_*oi*_ *− Q*_*ti*_) [17]. The analytical results based on CMT hold good agreement with the numerical simulations, as depicted in Fig. 4b.

**Fig. 5:**
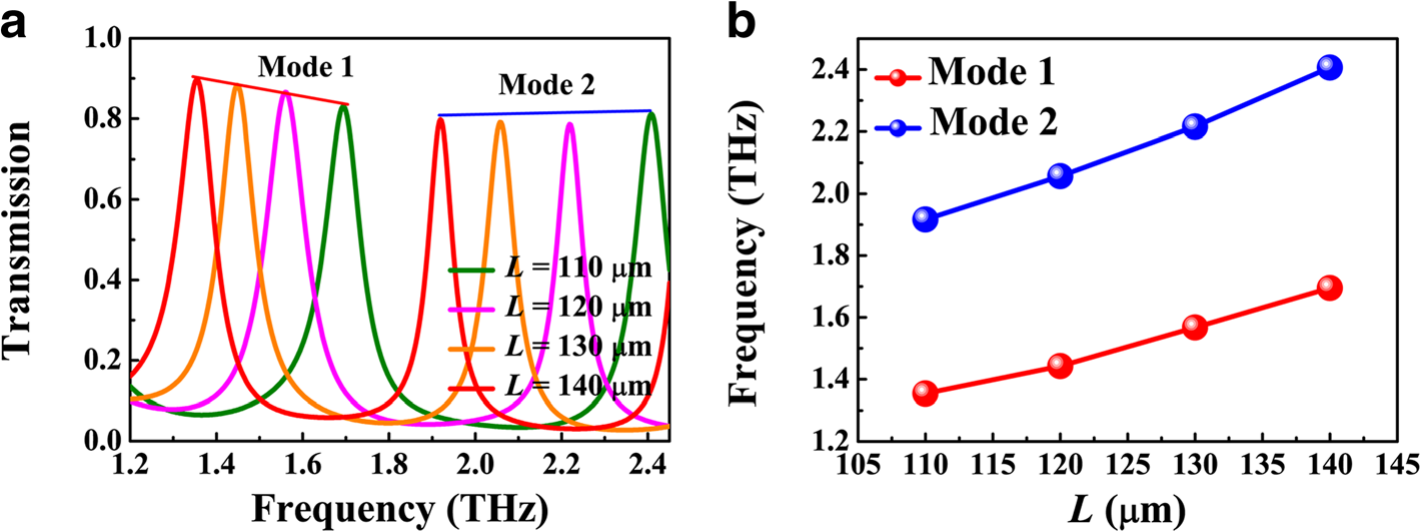
**a** Numerical transmission spectra for different cavity length *L*. **b** Resonant frequencies of modes 1 and 2 as functions of the cavity length *L*. Here, *g* = 1 μm, *d* = 5 μm, and *E*_F_ = 70 meV

**Fig. 6:**
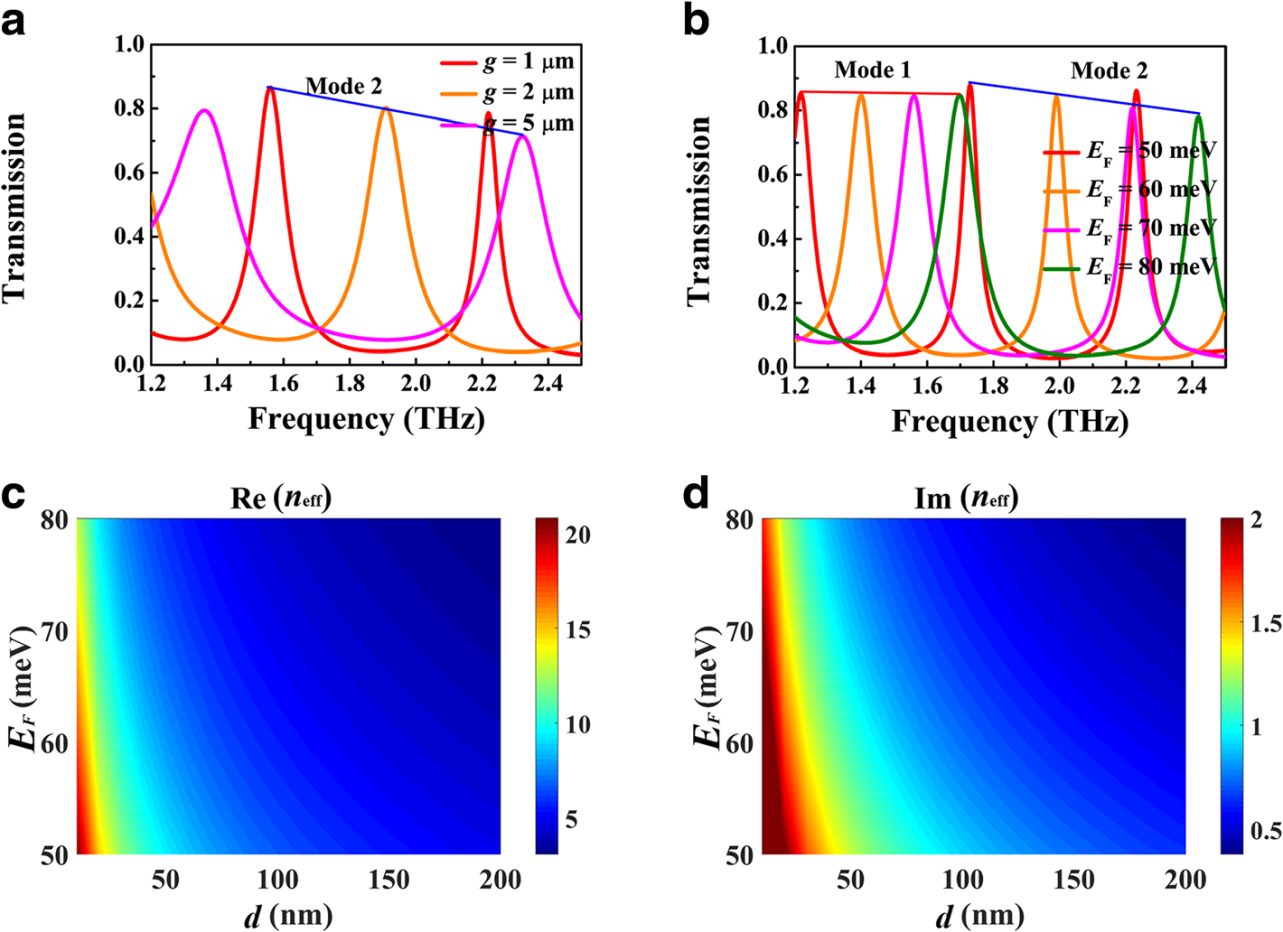
Transmission spectra for different gap width *g* (**a**) and Fermi energy *E*_F_ (**b**), where the other parameters are same as Fig. 4b. Dependence of Re(*n*_eff_) (**c**) and Im(*n*_eff_) (**d**) on the Fermi energy *E*_F_ and gap width *g*

Figure 5 shows the dependence of resonant frequency on the cavity length *L*, where *g* = 1 μm, *d* = 5 μm, and *E*_F_ = 70 meV. The transmission peak tends to red-shift with the increase of *L*, as presented in Fig. 5a, which can be further described by the standing wave resonance condition 2*k*_SPP_(*ω*_*r*_)*L + θ* = 2*mπ* (*m* = 1, 2, 3, ...), where *θ* is the reflective phase shift from the silicon-air interface and *k*_SPP_(*ω*_*r*_) is the wavevector of BIM waveguide at resonant frequency. As shown in Fig. 5b, the resonant frequencies of the first and second modes indeed exhibit a red shift with the increase of *L*. According to Eq. (1), the mode confinement is affected by gap width *g* which therefore have impact on the resonant frequency. Figure 6a presents the transmission spectra for different *g*, where *L* = 120 μm and *E*_F_ = 70 meV. With the increase of *g*, the resonant peak in same order exhibits a blue shift. This phenomenon can be attributed to the dramatic decrease of Re(*n*_eff_) as shown in Fig. 6c. The tuning of Fermi energy of BDS can be realized by alkaline surface doping in experiment. Figure 6b presents the transmission spectra for different Fermi energy, where the other parameters are same as Fig. 4b. As the Fermi energy increases, the transmission peak presents a blue shift, which can also be involved in the standing wave resonance picture. For a fixed length *L*, the FP cavity supports the resonance with defined SPP wavelength *λ*_SPP_ = *λ*_0_*/*Re(*n*_eff_), where *λ*_0_ is the incident wavelength. As shown in Fig. 6c, Re(*n*_eff_) is reduced with the increase of Fermi energy. As a result, the incident wavelength *λ*_0_ should be decreased as well to keep *λ*_SPP_ as a constant. That is the reason why the transmission peak tends to blue-shift with the increase of Fermi energy. Meanwhile, the bandwidth of the transmission peak is narrowed, which can be attributed to the decrease of Im(*n*_eff_), i.e., the propagation loss of the plasmonic waveguide mode in BIM waveguide.

## Conclusions

In summary, we have demonstrated the highly confined terahertz plasmonic mode supported by a BIM waveguide. The mode confinement and loss characteristics have been discussed with the variations of BDS-metal separation and Fermi energy, which indicates that there is an optimized frequency range with enhanced mode confinement as well as reduced propagation loss, which has rarely been reported in traditional SPP mode in metal structure. Different from previously studied BDS-based structure, the mode of this BIM waveguide can be efficiently supported in very narrow slit with width smaller than *λ*_0_/2000. By taking two silicon ribbons as reflective mirrors, a dynamically tunable band-pass filter has been achieved, where the resonant frequency can be actively controlled by adjusting the Fermi energy of BDS film without re-optimization of its structural parameters.

## Methods

Numerical results are obtained by using 2D finite-difference time-domain (FDTD) method, where the perfectly matched layers are set to absorb the scattering light in the *x* and *y* directions. The mesh size of the BDS film are set as d*x* × d*y* = 1 μm × 0.02 μm to achieve good convergence.

The frequency-dependent conductivity of BDS is described by Kubo formula with random phase approximation [12, 25].

3 $$ \operatorname{Re}\sigma \left(\Omega \right)=\frac{e^2}{\mathrm{\hslash}}\frac{tk_F}{24\pi}\Omega G\left(\Omega /2\right), $$

4 $$ \operatorname{Im}\sigma \left(\Omega \right)=\frac{e^2}{\mathrm{\hslash}}\frac{tk_F}{24{\pi}^2}\left\{\frac{4}{\Omega}\left[1+\frac{\pi^2}{3}{\left(\frac{T}{E_F}\right)}^2\right]+8\Omega {\int}_0^{\varepsilon_c}\left[\frac{G\left(\varepsilon \right)-G\left(\Omega /2\right)}{\Omega^2-4{\varepsilon}^2}\right]\varepsilon d\varepsilon \right\}, $$

where *G*(*E*) = *n*(−*E*) − *n*(*E*) and *n*(*E*) is the Fermi-Dirac distribution function, *E*_F_ is the Fermi energy of BDS, *k*_F_ = *E*_F_/*ћv*_F_ is its Fermi momentum, and *v*_F_ = 10^6^ m/s is the Fermi velocity. *ε* = *E/E*_F_, Ω = *ћω/E*_F_ *+ iћτ*^−1^*/E*_F_, where *ћτ*^−1^ = *v*_F_/(*k*_F_*μ*) is the electron scattering rate which shows strong dependence on the carrier mobility *μ. ε*_*c*_ = *E*_*c*_*/E*_F_ (*E*_*c*_ is the cutoff energy beyond which the Dirac spectrum is no longer linear), and *t* is the quantum degeneracy factor. Taking AlCuFe as an example, the fitting parameters in our calculations are set as follows: *t* = 40, *ε*_*c*_ = 3, *μ =* 3 × 10^4^ cm^2^ V^−1^ s^−1^ and *E*_F_ = 70 meV.

No human participants, data, or tissue or animals are involved in this research.

## Abbreviations

BDS: Bulk Dirac semi-metals
BIM: BDS-insulator-metal
CMT: Coupled mode theory
FDTD: Finite-difference time-domain
FWHM: Full width at half-maximum
SPPs: Surface plasmon polaritons
